# Two hymenopteran egg sac associates of the tent-web orbweaving spider, *Cyrtophoracitricola* (Forskål, 1775) (Araneae, Araneidae)

**DOI:** 10.3897/zookeys.874.36656

**Published:** 2019-09-02

**Authors:** Angela Chuang, Michael W. Gates, Lena Grinsted, Richard Askew, Christy Leppanen

**Affiliations:** 1 Department of Ecology and Evolutionary Biology, University of Tennessee, Knoxville, TN 37996, USA University of Tennessee Knoxville United States of America; 2 Systematic Entomology Laboratory, Beltsville Agricultural Research Center, Agricultural Research Service, US Department of Agriculture, c/o National Museum of Natural History, Smithsonian Institution, PO Box 37012, MRC-168, Washington, DC 20013-7012, USA Beltsville Agricultural Research Center Washington United States of America; 3 School of Biological Sciences, Royal Holloway University of London, Egham, Surrey, TW20 0EX, UK Royal Holloway University of London Egham United Kingdom; 4 Le Bourg est, St Marcel du Périgord, 24510 France Unaffiliated St Marcel du Périgord France

**Keywords:** colonial spider, Eulophidae, Eurytomidae, natural enemy, orbweaver

## Abstract

We report the discovery of two wasp species emerging from egg sacs of the spider *Cyrtophoracitricola* (Forskål 1775) collected from mainland Spain and the Canary Islands. We identify one as *Philolemapalanichamyi* (Narendran 1984) (Hymenoptera, Eurytomidae) and the other as a member of the *Pediobiuspyrgo* (Walker 1839) species group (Hymenoptera, Eulophidae). This is the first report of *Philolema* in Europe, and the first documentation of hymenopteran egg predators of *C.citricola*. The latter finding is particularly relevant, given the multiple invasive populations of *C.citricola* in the Americas and the Caribbean, where neither egg sac predation nor parasitism is known to occur. We describe rates of emergence by *Ph.palanichamyi* from spider egg sacs collected from the southern coast of Spain and estimate sex ratios and body size variation among males and females. We also re-describe *Ph.palanichamyi* based on the female holotype and male paratype specimens.

## Introduction

*Cyrtophoracitricola* (Forskål, 1775) is a widespread tent-web spider historically occurring in Mediterranean Europe, Asia, the Middle East, and across Africa (Forskål 1775; Kullmann 1958, 1959). Its distribution has burgeoned across the globe recently, with reported introductions in Colombia (Floréz-Daza 1996; Dossman et al. 1997; Levi 1997), the Dominican Republic (Alayón-García et al. 2001; Serra 2005), USA (Mannion et al. 2002), Cuba (Alayón-García 2003), Brazil (Álvares and De Maria 2004), Haiti (Starr 2005), Costa Rica (Víquez 2007), Jamaica (Crews et al. 2015), and other Caribbean islands (Armas 2010; Sewlal and Starr 2011).

The ecological impact of *C.citricola* in its new invasive ranges remains largely unknown; its colonial, group-living behavior results in wide-spanning networks of individual capture webs (Leborgne et al. 1998) that can swathe large areas of the trees and other plants where they build webs (Edwards 2006). This is why these spiders have been termed nuisances in Colombian coffee plantations and Dominican citrus trees, as well as general backyard pests in Florida (Cárdenas-Murillo et al. 1997; Serra 2005; Edwards 2006). Given ongoing range expansions of *C.citricola* in some of its invasive ranges (Sánchez-Ruiz and Teruel 2006; Martín-Castejón and Sánchez-Ruiz 2010), knowledge of natural enemies from its native range is of particular interest.

In this study, we report two hymenopteran species reared from *C.citricola* egg sacs collected from their native Spanish range. We reared the egg predator *Philolemapalanichamyi* (Narendran, 1984) from egg sacs collected from the Iberian Peninsula as well as Tenerife in the Canary Islands. We only found *Pediobius* sp., a member of the *pyrgo* (Walker, 1839) species group, in Tenerife egg sacs. It is a suspected hyperparasitoid; these parasitize primary parasitoids. *Philolemapalanichamyi* is one of several related species (formerly *latrodecti* species group of *Eurytoma*) that uses spider eggs as a larval host, whereas other species of *Philolema* attack insects as primary or secondary parasitoids (Noyes 2018).

The discovery of a *Philolema* species in Spain with *C.citricola* as a host is interesting for several reasons. Firstly, *Philolema* has not been documented in Europe, having previously been recorded with an Afrotropical, Neotropical, and Oriental distribution (van Noort 2019). Secondly, *Ph.palanichamyi* was originally recorded as an egg predator of *Cyrtophoracicatrosa* (Stoliczka, 1869) in India but has never been reported with *C.citricola* (Narendran 1994). In fact, only one other parasitoid of *C.citricola* has been previously reported: *Eurytomacyrtophorae* Zerova from Yemen (Zerova et al. 2008). It is clear from the illustrations and description that *E.cyrtophorae* belongs in *Philolema*, and is perhaps synonymous with *Ph.palanichamyi*. We were unable to compare our specimens with the type specimen of *E.cyrtophorae*, as it was unavailable for examination.

Members of the *Pe.pyrgo* species group are most often primary parasitoids of Lepidoptera or hyperparasitoids through primary hymenopteran parasitoids. Spider associations have been previously documented in this genus: Jamali et al. (2018) described *Pe.hebbalensis* Jamali, Zeya & Veenakumari, 2018 from an unidentified spider egg sac in India and Schoeninger et al. (2015) report *Pe.pyrgo* as a primary predator in egg sacs of *Latrodectusgeometricus* Koch, 1841 from Brazil. A series in the Smithsonian National Museum of Natural History (**USNM**) is labeled as reared from the egg sacs of *Latrodectus* “*scomotricus*”. This specific epithet does not apply to any described *Latrodectus* species and we suspect that this is a transcription error of “*geometricus*”, which is known from Florida (Pearson 1936).

Here, we describe our observations of *Ph.palanichamyi* emerging from *C.citricola* egg sacs in the wild and present preliminary data collected on the prevalence of egg parasitism, mean parasitoid emergence, and mean spiderling emergence in parasitized egg sacs from wild-collected egg sacs. We also report on the morphological variation within both sexes, provide mean body size measurements, and describe sex ratio variation. In laboratory settings we test whether wasps can infect *C.citricola* egg sacs without intermediate hosts. Importantly, we also re-describe the female holotype and for the first time a male paratype of *Ph.palanichamyi*.

## Materials and methods

**Figures 1–2.**
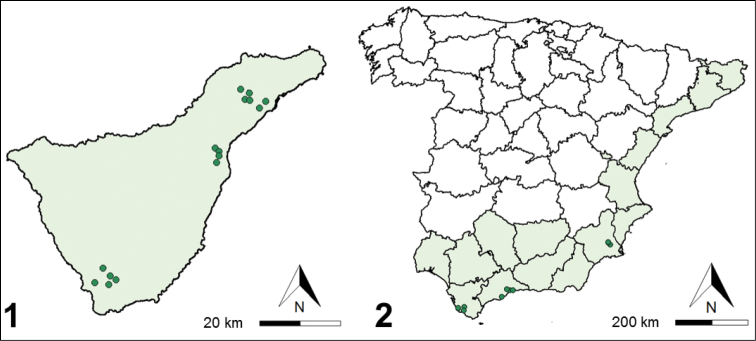
Points indicate where *Ph.palanichamyi* was found in **1** Tenerife, Canary Islands as well as **2** along the southern coast of Spain. *C.citricola* is known from the shaded regions (Cardoso and Morano 2010). *Pe.pyrgo* was only found at Tenerife locations.

**Figures 3–5.**
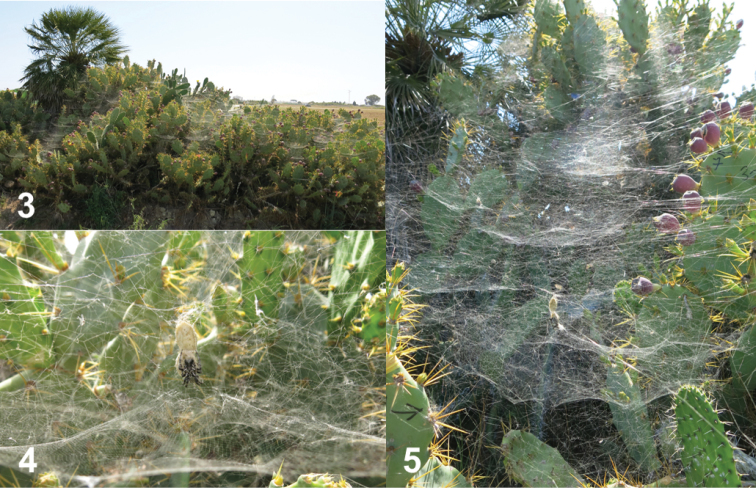
Representative *C.citricola* colonies in mainland Spain and the Canary Islands where *Ph.palanichamyi* and *Pe.pyrgo* were observed or collected.

We hand-collected *C.citricola* egg sacs between 30 May and 16 June 2016 and between 4 October and 1 November 2018 on the Iberian Peninsula. These areas experience hot summer Mediterranean (Cádiz and Málaga provinces) and cold semi-arid steppe (Murcia and Valencia provinces) Köppen climates of south and east coastal Spain. We further collected egg sacs from Tenerife, Canary Islands, between 29 May and 16 June 2018, in mountainous habitats of the north and dry, scrub habitats of the south (Figs 1, 2). In all areas, we primarily found *C.citricola* colonies in sun-exposed habitats with non-native succulents, such as *Opuntia* spp. (Cactaceae), *Austrocylindropuntia* spp. (Cactaceae), and *Agave* spp. (Asparagaceae) (Chuang and Leppanen 2018). These were most commonly identified to *Opuntiaficusindica* L. (Mill.), *Austrocylindropuntiasubulata* (Muehlenpf.) Backeb., and *Agaveamericana* L. (Deltoro, personal communication) and found in dry, grass-dominated habitats by roadsides or cultivated on rural private property (Figs 3–5).

In the field, we transferred egg sacs to 59.1 mL clear polypropylene containers with clear polyethylene lids. These were then transported to the University of Tennessee (Knoxville, Tennessee, USA) and stored in the laboratory at 21.0–23.5 °C and a 14 (light): 10 (dark) hour photoperiod. We misted the egg sacs weekly with water for up to eight weeks after the collection period. The wasps were confined to cups as they emerged, and we killed them by freezing and made post-mortem wasp counts and egg sac dissections afterwards.

We estimated *Ph.palanichamyi* variation in body size and sex ratio from those individuals reared from 11 egg sacs. In two cases, two egg sacs were conjoined and the wasps emerged from each of the pairs into their one shared container. The remaining seven egg sacs were kept in seven separate containers. Hence, we used nine batches of wasps, seven originating from single egg sacs and two from pairs of egg sacs. After we froze them, wasps and egg sacs were allowed to air-dry. We dissected egg sacs by teasing the looser silk-domed surface from the firmer silk flat ‘floor’. All of the wasps that had emerged, or were still inside the egg sac, were sexed and measured provided that they were in suitable condition. Wasps without a gaster or that had failed to emerge completely from the pupal case were not measured. We measured the distance from the front of the pronotum to the tip of the metasoma to estimate body size using a squared graticule in a microscope eyepiece at uniform magnification. The head was omitted from the body length measurement because it had become detached from a number of specimens. In total, 665 wasps were sexed and 576 were measured.

To test whether *Ph.palanichamyi* reared in the laboratory could infect *C.citricola* egg sacs, we placed a freshly laid egg sac (produced under laboratory conditions) in a container with multiple adult male and female wasps. After seven days, we removed the egg sac and placed it in a separate container at room temperature (20±2 °C) for four weeks after which we carefully opened the egg sac to reveal its contents.

For specimen preparation, we preserved the wasps in 80% ethanol and dehydrated them through increasing concentrations of ethanol before transferring them to hexamethyldisilazane (HMDS) (Heraty 1998) for point-mounting. We used a Leica 205c stereomicroscope with 10X oculars and a Leica LED ring light source for point-mounted specimen observation.

We took scanning electron microscope (SEM) images with a Hitachi TM3000 (Tungsten source). We adhered body parts of disarticulated specimens to a 12.7 × 3.2 mm Leica/Cambridge aluminum SEM stub by a carbon adhesive tab (Electron Microscopy Sciences, #77825-12). We used a Cressington Scientific 108 Auto to sputter coat stub-mounted specimens with gold-palladium from multiple angles to ensure complete coverage (~20–30 nm coating). To capture the habitus image of the holotype and recently reared female we used a Macropod Pro 3D system (Canon 6D Mark II body) with a Canon EF 70-200 mm telephoto with affixed 10× objective lens (Macroscopic Solutions, LLC). Our image series were merged into a single in-focus, composite image with the program Zerene Stacker (ver. 1.04). Post-imaging processing was completed with built-in editing tools in Zerene Stacker, Photoshop CS4 and InDesign CS5.

Specimens from the Smithsonian Institution National Museum of Natural History and borrowed holotypes of *Ph.palanichamyi* and *Ph.lankana* Narendran, 1994 were compared with our reared specimens by MG. RA provided independent confirmation of chalcidoid identity. We used keys in Bouček (1965) and Cao et al. (2017) for determining *Pediobius* and deposited specimens reared as part of this study in the National Museum of Natural History, Washington, DC.

The hymenopteran terminology we use for surface sculpture follows Harris (1979) and for morphology follows Gibson et al. (1997). Fu is used as an abbreviation for funicular segment, Gt_n_ for gastral tergum, and Gs_n_ for gastral sternum. We took several measurements, including the following: body length, in lateral view from the anterior projection of the face to the tip of the metasoma; head width through an imaginary line connecting the farthest lateral projection of the eyes; head height through an imaginary line from the vertex to the clypeal margin bisecting both the median ocellus and the distance between the toruli; malar space, in lateral view between the ventral margin of the eye and lateral margin of the oral fossa; posterior ocellar line (POL), the shortest distance between the posterior ocelli; ocular ocellar line (OOL), the shortest distance between the lateral margin of the posterior ocellus and the eye orbit; posterior ocellar diameter (POD), the longest diameter of the posterior ocellus; marginal vein, the length coincident with the leading forewing edge to the base of the stigmal vein; stigmal vein, the length between its base on the marginal vein and its apex; and postmarginal vein, the length from the base of the stigmal vein to its apex on the leading forewing edge. We measured the mesosomal sclerites and metasomal terga dorsally along the midline. **LS** stands for multiporous plate sensilla; wing venation abbreviations are **MV** (marginal), **PMV** (postmarginal), and **STG** (stigmal).

We use the following abbreviations for collections: **USNM** (National Museum of Natural History, Smithsonian Institution, Washington, DC, USA) and **BMNH** (The Natural History Museum, London, England).

## Results

### Field and laboratory observations

#### *Philolemapalanichamyi* emergence rates

In the field, we observed *Ph.palanichamyi* emerging from *C.citricola* egg sacs on 11 June 2016 in Málaga, Andalusia. Owing to the central location of the egg sacs within a web, dozens of these wasps were immediately snared in the surrounding web or consumed by nearby *C.citricola* colony members.

*Philolemapalanichamyi* emerged from 43 of 103 groups of 1–5 (mean = 1.86 ± 0.11) conjoined egg sacs collected in Cádiz (21 of 37 groups), Málaga (14 of 21 groups), Murcia (8 of 34 groups), but not in Valencia (0 of 11 groups) (Figs 1, 2; Table 1). A mean of 56.7 ± 11.4 (median = 43) wasps emerged from 18 singly confined egg sacs. A mean of 38.6 ± 22.2 (median = 13) spiderlings emerged in 7 of 18 singly confined egg sacs with wasps and 102.4 ± 21.0 (median = 117) in 18 of 34 egg sacs without wasps.

**Table 1.** Parasitism rate by *Ph.palanichamyi* for each of four collection locations. We describe parasitism rate per string of egg sacs ranging from 1–5 egg sacs per female.

*Ph.palanichamyi* parasitism of *C.citricola* egg sacs by location			
Location	# Egg sac strings		Parasitism rate (%)
	With wasps	Total	
Cádiz	21	37	56.8
Málaga	14	21	66.7
Murcia	8	33	24.2
Valencia	0	12	0
Total	43	103	41.7

#### *Philolemapalanichamyi* body sizes and sex ratios

**Figure 6.**
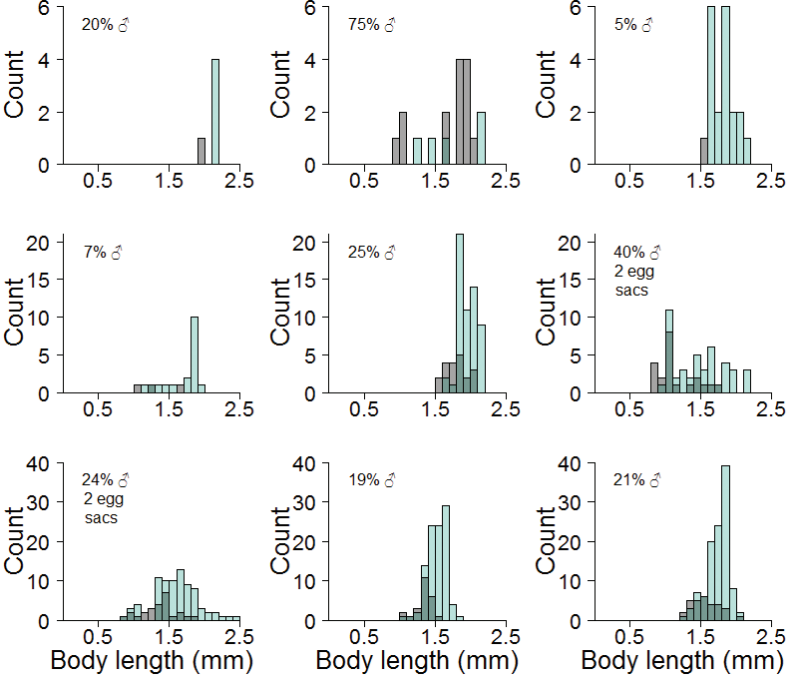
Histograms showing the distribution of body sizes of females (green) and males (grey; any overlap between males and females is greyish green) from 9 batches of wasp emergences: 7 batches from single egg sacs and 2 batches from pairs of egg sacs.

We sexed 665 adults from 11 egg sacs. Between 5 and 151 wasps emerged from single egg sacs (median = 59; mean = 67.29). Wasps emerged from up to eight exit holes made without an observed preference in either the domed surface or the silken floor of the egg sac. The sex ratio varied greatly among egg sacs from 5.0% to 75.0% males (median = 20.0%; mean 24.6% males) (Table 2). Of the 576 adults measured, females were slightly larger than males, although both male and female body sizes varied greatly both within and among egg sacs (Fig. 6): mean female body size was 1.68 mm (median = 1.69 mm; range: 0.84–2.41mm) while mean male body size was 1.47 mm (median = 1.44 mm; range: 0.80–2.07 mm). It should be noted that ‘body size’ is less than the total body length because the head was not included in the measurement.

**Table 2.** Female and male average lengths and sex ratios for each group of measured egg sacs.

ID	# Egg sacs	# Females	# Males	# Wasps	Sex ratio (% males)	Female av. length	Male av. length
1	1	4	1	5	20	2.16	1.98
2	1	5	15	20	75	1.71	1.69
3	1	21	1	22	4.5	1.82	1.56
4	1	55	4	59	6.8	1.71	1.29
5	1	60	20	80	25	1.97	1.8
6	2	50	33	83	39.8	1.47	1.14
7	2	84	26	110	23.6	1.6	1.35
8	1	109	25	134	18.7	1.52	1.35
9	1	119	32	151	21.2	1.75	1.57

A variable primary sex ratio (i.e., that of deposited eggs) that favors females is usual in Chalcidoidea, enabled by their haplodiploidy. Several factors can influence this ratio in favor of an increased proportion of males (unfertilized eggs), including smaller hosts, less quality hosts, or more numerous hosts at increased density (Godfray 1994). None of these effects are apparent in our limited sample. Older female wasps may suffer a diminishing supply of stored sperm so that increasing numbers of unfertilized eggs are laid. This is one possible explanation for the brood that was 75% male. It is also possible that smaller males may be favored when larval food is limited.

### Parasitism under laboratory conditions

When we opened the egg sac presented in the laboratory to adult *Ph.palanichamyi*, several live wasp larvae were visible, confirming the association of *Ph.palanichamyi* with *C.citricola*. Furthermore, each larva appeared considerably larger than a single spider egg, suggesting that a single larva might feed on multiple eggs within the egg sac. This and the lower ratio of wasps to spiderlings found in parasitized compared with unparasitized egg sacs also suggests that this species is an egg predator, not an egg parasitoid. The first wasp offspring eclosed from its pupa seven weeks after the fresh egg sac was introduced to adult wasps.

## Taxonomy

### Philolema palanichamyi

Taxon classificationAnimaliaHymenopteraEurytomidae

Narendran

9A480597130D5328976CEB2F801CE3CA

Figs 7 –16

#### Re-description.

**Figures 7–8.**
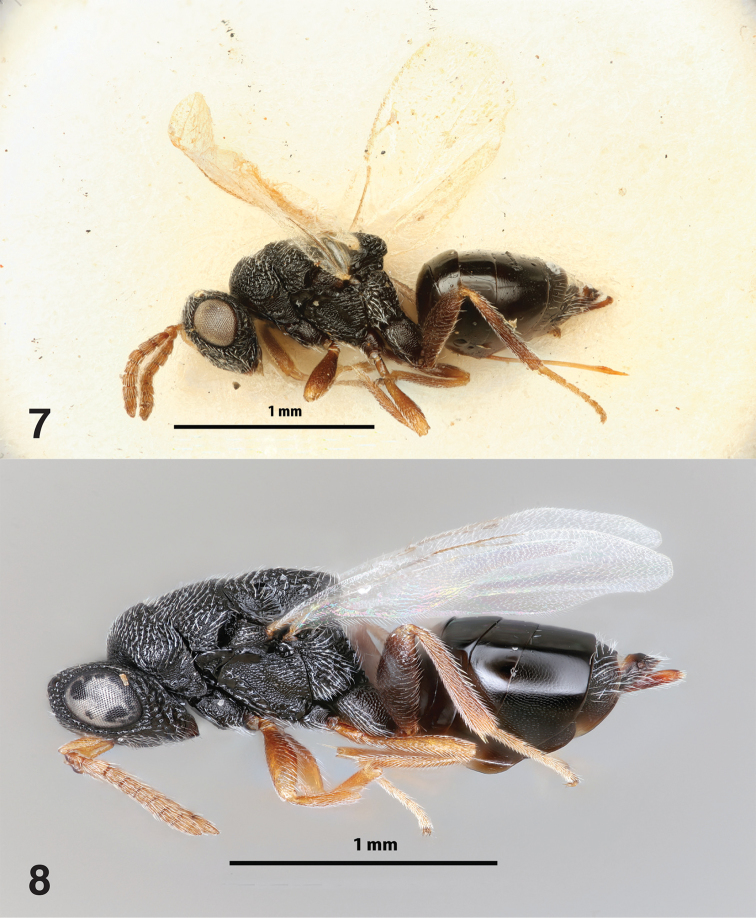
*Philolemapalanichamyi***7** female holotype **8** female habitus. Scale bars: 1 mm.

Based on female holotype (Fig. 7; Fig. 8: female from specimens reared during this study, not used in re-description). Length 2.2 mm. Body black except the following: scape, pedicel, apex femur, apex and base tibia (yellowish brown to brown), tarsus (golden), flagellum, mid coxa, femur, and tibia (brown). Ovipositor sheaths brown. Wings hyaline, setation pale. Venation golden.

**Figures 9–16.**
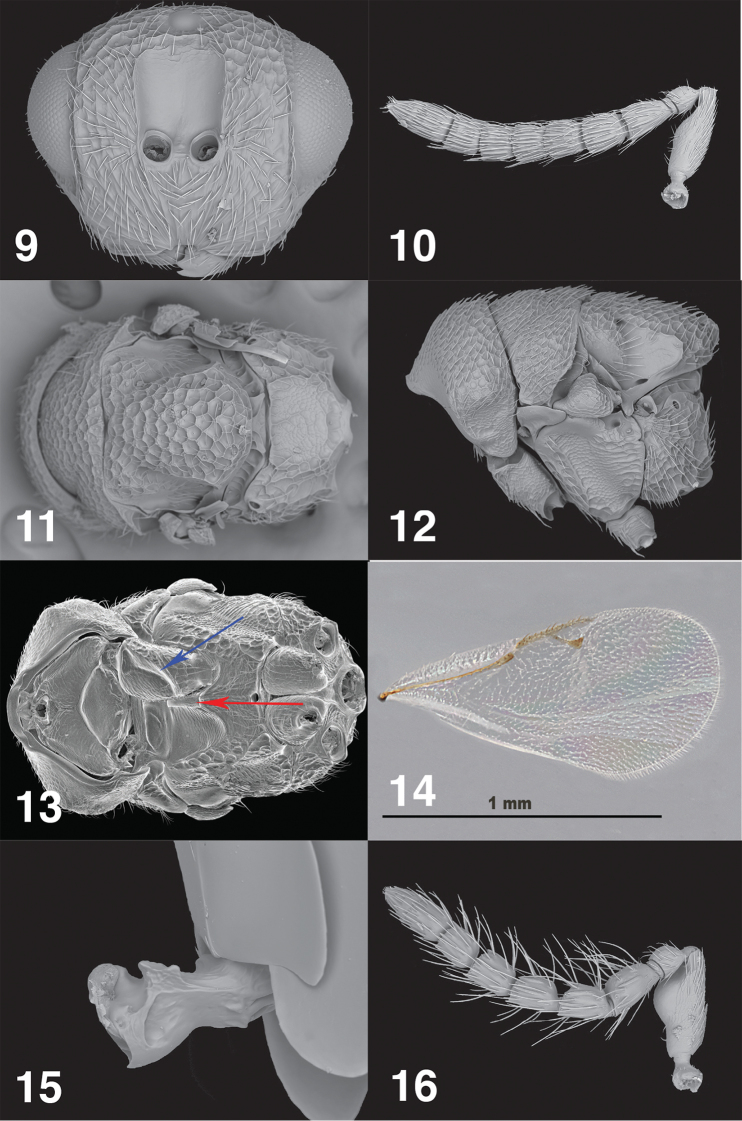
*Philolemapalanichamyi***9** female, anterior head **10** female antenna **11** female dorsal mesosoma **12** female mesosoma, lateral **13** female ventral mesosoma **14** female fore wing **15** female petiole **16** male antenna.

***Head*** (Fig. 9). 1.43× as broad as long, broader than mesosoma. Eye 1.08× as long as malar space. POL 2.0× as large as OOL; the latter 1.88× as large as POD. Malar space 0.93× as long as width of oral fossa and equal to height of eyes. Distance between toruli 1.0× their own diameter. Adscrobal area subequal in width to acarinate antennal scrobes. Lower face striate, clypeus emarginate. Malar space with groove in dorsal half below eye, continuous with striation. Gena striate to umbilicate.

***Antenna*** (Fig. 10). Scape linear, 3.0× as long as broad. Pedicel short, 1.29× as long as broad. Funicle 6-segmented, setae decumbent. Fu_1_ 1.30× as long as broad, Fu_2_ just longer than broad, Fu_3–5_ quadrate. Funiculars with single row LS. Clava 2-segmented, 1.80× as long as broad; bearing also the same pattern of LS as for the funiculars; segments fused, bearing two rows of LS.

***Mesosoma*** (Figs 11–13). 1.71× as long as broad. Pronotal collar 3.60× as broad as long; mesoscutum 1.25× as broad as long; mesoscutellum 1.33× as long as broad. Mesosoma dorsally umbilicate, interstices coriaceous. Notauli shallow, crenulate. Puncturation of mesoscutellum somewhat sparser than that of mid lobe of mesoscutum; mesoscutellum overhanging postscutellum. Axillar grooves crenulate, shallow. Postscutellum punctured mesally. Propodeum sloping at an angle of about 80° with main axis of mesosoma, evidently convex from side to side, with incomplete areolate stripe mesally delimited submedian ridges, on either side irregularly areolate; setation fine, erect and proclinate between the spiracles, dense very long and reclinate laterally; spiracle elliptic at posterior margin of metanotum. Tegula umbilicate. Prepectus with lateral panel glabrous. Mesopectus anteriorly depressed as scrobes to receive forecoxae, mesodiscrimen produced as beak-like prominence (Fig. 13, red arrow), scrobes glabrous anteriorly becoming coriaceous, adscrobal area umbilicate dorsally, coriaceous below; mesepisternum with femoral scrobe finely and densely reticulate; mesepimeron reticulate-carinate on ventral 1/3, with longitudinal carinae in dorsal 2/3. Metepimeron umbilicate, setae dense and long. Mesotrochantinal plate entirely sclerotized, its anterior carinate margin emarginate at mesofurcal pit. Metepisternum with lateral lobes anterad metacoxal foramina that overhang metafurcal pits.

***Legs*** (Fig. 13). Procoxa depressed anteriorly with diagonal carina delimiting depression (Fig. 13, blue arrow). Mesocoxa without lamella. Metacoxa bare dorsobasally, mostly coriaceous to finely reticulate.

***Forewing*** (Fig. 14). Setation fine and pale making wing appear sparsely setose, MV:PMV:STG as 20:25:19. Stigma with line of four sensilla placodea; parastigma bearing 3 adjacent sensilla placodea forming a triangle. Cubital and basal folds setose; basal cell with 3–6 irregularly distributed setae.

***Petiole*** (Fig. 15). In dorsal view just longer than broad, carinate anteriorly and produced anterolaterally as angulate processes, surface rugulose.

***Gaster.*** Smooth dorsally, very faintly alutaceous laterally, just shorter than mesosoma, Gt4 longest tergum, ~2.0× as long as Gt_3_. Gt_1_ and Gt_2_ asetose, Gt_3_ and Gt_4_ with a few setae dorsolaterally [some appear to have been abraded], Gt_5_ and Gt_6_ and syntergum more densely setose.

**Figures 17–19.**
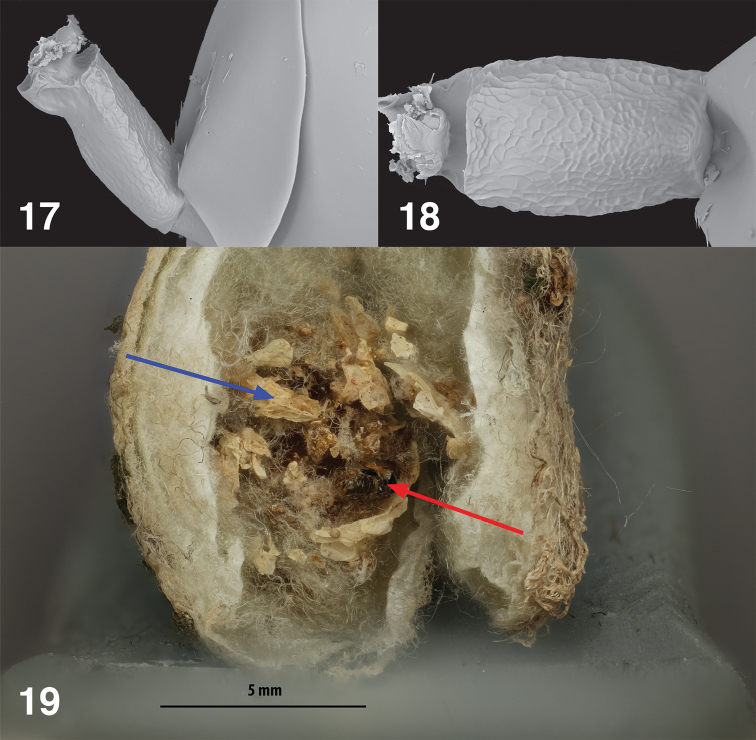
*Philolemapalanichamyi***17** male petiole, lateral **18** male petiole, dorsal **19** Dissected *C.citricola* egg sac, blue arrow = dried, consumed spider eggs, red arrow = dead *Ph.palanichamyi*.

**Male** (Figs 16–18). Similar to female in color and sculpture, differing in form of antenna and metasoma as below.

***Antenna*** (Fig. 16). Scape 2.11× as long as broad, with smooth surface on protuberant ventral plaque bearing pores (visible under high magnification only) on apical two thirds. Pedicel 1.50× as long as broad. Funicle 5-segmented with funiculars progressively shortening, each funicular with two whorls of long setae and a single row of LS visible; F_1–3_ asymmetric themselves progressively shortening; Fu_4–5_ symmetric, subquadrate. Clava 2-segmented, 2.30× as long as broad.

***Petiole*** (Figs 17, 18). 1.66× as long as broad, slightly carinate anteriorly, its surface reticulate.

***Gaster.*** Smooth dorsally, very faintly alutaceous laterally, somewhat shorter than mesosoma, Gt4 longest tergum, ~1.25× as long as Gt3. Gt1 and Gt2 asetose, Gt3 and Gt4 with a few setae dorsolaterally [some appear to have been abraded], Gt5 and Gt6 and syntergum more densely setose.

***Variation.*** Specimens vary greatly in size from approximately 0.8–2.5 mm total length. Prominence and extent of morphological characters diminishes with decreasing body size; for example, general body sculpture is less apparent in small specimens.

#### Material examined.

**Holotype**, ♀: INDIA: Timadu, Palani, 1992, coll. Palanichamy, Host *Cyrtophoracicatrosa* (spider); **holotype**, *Desantiscapalanichamyi* ♀, sp. nov., det. Narendran 1983; B.M. TYPE HYM 5.3060; NHMUK013455729 (BMNH). **Paratype**, ♂: INDIA: Timadu, Palani, 1992, coll. Palanichamy, Host *Cyrtophoracicatrosa* (spider); **paratype**; *Desantiscapalanichamyi* ♂ sp. nov., det. Narendran 1983 (USNM). **Other material**, SPAIN: Murcia: Murcia, 5.VI.2016, 37.9176N -1.20633W, A. Chuang, Lot #593 (88 ♀♀, 15 ♂♂), Lot#971-16 (6 ♀♀); Málaga: Málaga, 8.VI.2016, 36.73705N -4.40486W, A. Chuang, Lot #609-1 (108 ♀♀, 25 ♂♂), Lot#956-16 (7 ♀♀), Lot #615 (16 ♀♀, 3 ♂♂); Cádiz: Cádiz, 13.VI.2016, 36.31301N -5.8865W, A. Chuang, Lot #627-1 (27 ♀♀, 4 ♂♂); Cádiz, 16.VI.2016, 36.29552N -6.0748W, A. Chuang, Lot #637-4 (14 ♀♀, 2 ♂♂), Lot#926-1 (8 ♀♀, 1 ♂); Tenerife: 28.07608N -16.6483W, Lot#899-A (15 ♀♀, 4 ♂). All deposited in USNM.

#### Recognition.

This species can be distinguished from the widespread *Philolemalatrodecti* Fullaway, 1953 by the suberect flagellar setation (females only, adpressed in *Ph.palanichamyi*) and smaller ventral plaque (males only, less than half the depth seen in *Ph.palanichamyi*). Usually, the sculpture of the tegula is much more distinct in *Philolemalatrodecti*. Also, *Ph.palanichamyi* is known only from the eggs of *Cyrtophora* spp. while *Ph.latrodecti* is known only from the eggs of *Latrodectus* spp.

#### Biology.

Egg predator (Fig. 17) of *Cyrtophora* spp. (Araneae, Araneidae).

##### Pediobius species group pyrgo

We examined 4♀♀ and 3♂♂ of a *Pediobius* species reared from *C.citricola* egg sacs collected by AC on Tenerife in the Canary Islands in May 2018. These specimens closely resemble material identified as *Pe.pyrgo* (Walker) from England and elsewhere in Europe reared from lepidopteran hosts as primary or very often secondary parasitoids. Only small and probably insignificant differences could be found. It seems best to regard the *Pediobius* material reared from *Cyrtophora* egg sacs collected in Tenerife as probably *Pe.pyrgo* until more material is available for morphological and molecular analyses.

As currently understood, *Pe.pyrgo* has been reported from an unusually broad range of primary hosts (Noyes 2018) that it attacks directly or as a facultative hyperparasitoid. Lepidoptera are the most frequently recorded hosts, but Dermaptera, Diptera, and Hymenoptera have also been reported. Larvae or pupae from thirteen families of Lepidoptera, including leaf-miners, web spinners, case-bearers, as well as exposed feeders are known as hosts. However, in many instances it is their parasitoids that are attacked by *Pe.pyrgo*, especially ichneumonoid Hymenoptera but also other chalcidoids (Eulophidae, Pteromalidae).

*Pediobiuspyrgo* is a solitary or slightly gregarious endoparasitoid of larvae and pupae, and it has been described as a koinobiont larva/pupal parasitoid of *Leucoptera* (Lep., Lyonetiidae) (Mey 1993). It is widespread in the Palaearctic and Oriental regions with a few New World records from North, South, and Central America. Schoeninger et al. (2015) record *Pe.pyrgo* as associated with *Latrodectus* eggs sacs in South America, and *Pe.brachycerus* (Thomson) and a few other species of *Pediobius* are known to be associated with spider egg sacs (Bouček 1965; Noyes 2018), but these do not belong to the species group *pyrgo*.

## Discussion

Although egg parasitism or predation in spiders has received little attention, case studies suggest it may be common in native ranges (e.g., van Wingerden 1973; Rollard 1985; van Baarlen et al. 1994; Dinter 1996; Sacher 2001; Finch 2005; Leborgne and Pasquet 2005; Krehenwinkel et al. 2016; Wawer and Kostro-Ambroziak 2016), resulting in high mortality rates in parasitized egg sacs (Finch 2005; Krehenwinkel et al. 2016; Wawer and Kostro-Ambroziak 2016). Smith (1982) documented fewer offspring of the orbweaver spider *Philoponellaoweni* (Chamberlin 1924) owing to parasitism by the pteromalid wasp *Arachnopteromalusdasys* Gordh, 1976. Hesse reported that *Philolemaarachnovora* (Hesse 1942) appears to be an egg predator of *Latrodectusindistinctus* Pickard-Cambridge, 1904, consuming more than one egg per wasp larva. This was based on dissections of parasitized egg sacs, similar to our own findings.

Overall, we found that *Ph.palanichamyi* was present in about 40% of egg sacs. While wasp presence did not completely preclude spiderling emergence, it was associated with about 60% fewer spiderlings. It thus seems likely that *C.citricola* eggs in Spain experience predation pressure from the wasplarvae, an issue that is ripe for more detailed examination. Additionally, it would be useful to understand whether wasp predation rates remain stable across *C.citricola*’s range and breeding season and whether they promote extinction patterns in this spider, as is well-known among spider colonies (Avilés 1997).

The discovery of these two hymenopteran associates of *C.citricola* in its native Spanish range has particularly important implications for the multiple introductions of this spider throughout the Americas and Caribbean. No wasp associates have been reported from any of the non-native populations of *C.citricola*, even though yearly surveys of two expanding populations in Florida from 2014–2017 have been conducted by AC (Chuang, unpublished data). This is notable because in Florida, the range of *C.citricola* overlaps *L.geometricus* and its egg sac parasitoid *Ph.latrodecti*, known only to parasitize the widow spiders *Latrodectrus* spp. (Bibbs and Buss 2012) and with no evidence of a host shift to *C.citricola*. The introduction of *Ph.palanichamyi* may thus have effects on the population dynamics, range expansion, and impacts of non-native *C.citricola* spiders.

## Supplementary Material

XML Treatment for Philolema palanichamyi

## Supplementary materials

Supplementary material 1 Table S1. Egg predator and spiderling emergence rates Data type: species data https://binary.pensoft.net/file/329925This dataset is made available under the Open Database License (http://opendatacommons.org/licenses/odbl/1.0/). The Open Database License (ODbL) is a license agreement intended to allow users to freely share, modify, and use this Dataset while maintaining this same freedom for others, provided that the original source and author(s) are credited.Angela Chuang, Michael W. Gates, Lena Grinsted, Richard Askew, Christy Leppanen
