# Supplementary material for: Two hymenopteran egg sac associates of the tent-web orbweaving spider, Cyrtophoracitricola (Forskål, 1775) (Araneae, Araneidae)
Source: Zookeys. 2019 Sep 2;874:1–18. doi: 10.3897/zookeys.874.36656 (PMC6733805; doi:10.3897/zookeys.874.36656)
Supplement: Supplementary material 1 [file zookeys-874-001-s001.docx]

Supplementary Table 1. Egg predator and spiderling emergence rates

| Collection date | Location | Latitude | Longitude | # Egg sacs in chain | Wasp emergence | *Ph. palanichamyi* count | Spiderling emergence | Spiderling count | Wasp and spiderlings both present |
| --- | --- | --- | --- | --- | --- | --- | --- | --- | --- |
| 5/30/2016 | Valencia | 39.57778 | -0.43648 | 1 | No | 0 | No | 0 | No |
| 5/30/2016 | Valencia | 39.57886 | -0.43844 | 2 | No | 0 | No | 0 | No |
| 6/1/2016 | Valencia | 39.6141 | -0.29468 | 1 | No | 0 | Yes | 190 | No |
| 6/1/2016 | Valencia | 39.6141 | -0.29449 | 1 | No | 0 | Yes | 7 | No |
| 6/1/2016 | Valencia | 39.61411 | -0.30264 | 1 | No | 0 | Yes | 27 | No |
| 6/1/2016 | Valencia | 39.61411 | -0.30264 | 1 | No | 0 | No | 0 | No |
| 6/1/2016 | Valencia | 39.61411 | -0.30264 | 1 | No | 0 | Yes | 1 | No |
| 6/1/2016 | Valencia | 39.61411 | -0.30264 | 1 | No | 0 | No | 0 | No |
| 6/1/2016 | Valencia | 39.61411 | -0.30264 | 1 | No | 0 | Yes | 115 | No |
| 6/1/2016 | Valencia | 39.61411 | -0.30264 | 1 | No | 0 | No | 0 | No |
| 6/3/2016 | Valencia | 39.62514 | -0.33122 | 2 | No | 0 | No | 0 | No |
| 6/4/2016 | Murcia | 37.87013 | -1.14215 | 4 | Yes | 58 | No | 0 | No |
| 6/4/2016 | Murcia | 37.87013 | -1.14215 | 3 | No | 0 | No | 0 | No |
| 6/4/2016 | Murcia | 37.87013 | -1.14215 | 4 | No | 0 | Yes | 2 | No |
| 6/4/2016 | Murcia | 37.87013 | -1.14215 | 1 | No | 0 | Yes | 6 | No |
| 6/4/2016 | Murcia | 37.87014 | -1.14218 | 4 | Yes | 1 | No | 0 | No |
| 6/4/2016 | Murcia | 37.87014 | -1.14218 | 2 | No | 0 | No | 0 | No |
| 6/4/2016 | Murcia | 37.87015 | -1.14216 | 5 | Yes | 130 | Yes | 6 | Yes |
| 6/4/2016 | Murcia | 37.87015 | -1.14216 | 4 | No | 0 | Yes | 1 | No |
| 6/4/2016 | Murcia | 37.87015 | -1.14216 | 2 | No | 0 | Yes | 3 | No |
| 6/4/2016 | Murcia | 37.87019 | -1.14226 | 1 | No | 0 | No | 0 | No |
| 6/4/2016 | Murcia | 37.87019 | -1.14226 | 1 | No | 0 | No | 0 | No |
| 6/4/2016 | Murcia | 37.87019 | -1.14226 | 2 | No | 0 | No | 0 | No |
| 6/4/2016 | Murcia | 37.87019 | -1.14226 | 4 | No | 0 | No | 0 | No |
| 6/4/2016 | Murcia | 37.87019 | -1.14226 | 3 | No | 0 | Yes | 3 | No |
| 6/4/2016 | Murcia | 37.87019 | -1.14226 | 4 | No | 0 | No | 0 | No |
| 6/5/2016 | Murcia | 37.9176 | -1.20633 | 1 | Yes | 91 | No | 0 | No |
| 6/5/2016 | Murcia | 37.92572 | -1.20954 | 1 | Yes | 11 | Yes | 32 | Yes |
| 6/5/2016 | Murcia | 37.92572 | -1.20954 | 1 | Yes | 46 | Yes | 12 | Yes |
| 6/5/2016 | Murcia | 37.92572 | -1.20954 | 2 | Yes | 9 | Yes | 8 | Yes |
| 6/5/2016 | Murcia | 37.92572 | -1.20954 | 1 | Yes | 3 | Yes | 5 | Yes |
| 6/5/2016 | Murcia | 37.92572 | -1.20954 | 1 | No | 0 | No | 0 | No |
| 6/5/2016 | Murcia | 37.92572 | -1.20954 | 1 | No | 0 | No | 0 | No |
| 6/5/2016 | Murcia | 37.92572 | -1.20954 | 2 | No | 0 | Yes | 4 | No |
| 6/5/2016 | Murcia | 37.92572 | -1.20954 | 3 | No | 0 | No | 0 | No |
| 6/6/2016 | Murcia | 37.82881 | -1.21445 | 1 | No | 0 | No | 0 | No |
| 6/6/2016 | Murcia | 37.829 | -1.21424 | 2 | No | 0 | Yes | 214 | No |
| 6/6/2016 | Murcia | 37.83134 | -1.15964 | 1 | No | 0 | No | 0 | No |
| 6/7/2016 | Murcia | 37.91732 | -1.20651 | 1 | No | 0 | Yes | 1 | No |
| 6/7/2016 | Murcia | 37.91732 | -1.20651 | 0 | No | 0 | No | 0 | No |
| 6/7/2016 | Murcia | 37.91732 | -1.20651 | 1 | No | 0 | No | 0 | No |
| 6/7/2016 | Murcia | 38.05721 | -1.16533 | 1 | No | 0 | Yes | 3 | No |
| 6/7/2016 | Murcia | 38.05724 | -1.16514 | 1 | No | 0 | No | 0 | No |
| 6/7/2016 | Murcia | 38.05724 | -1.16514 | 3 | No | 0 | Yes | 114 | No |
| 6/7/2016 | Murcia | 38.05724 | -1.16514 | 2 | No | 0 | Yes | 222 | No |
| 6/8/2016 | Málaga | 36.57265 | -4.65656 | 1 | Yes | 2 | Yes | 168 | Yes |
| 6/8/2016 | Málaga | 36.57265 | -4.65656 | 1 | Yes | 43 | No | 0 | No |
| 6/8/2016 | Málaga | 36.57265 | -4.65656 | 2 | Yes | 18 | No | 0 | No |
| 6/8/2016 | Málaga | 36.57265 | -4.65656 | 2 | No | 0 | No | 0 | No |
| 6/8/2016 | Málaga | 36.57265 | -4.65656 | 1 | No | 0 | No | 0 | No |
| 6/8/2016 | Málaga | 36.57265 | -4.65656 | 1 | No | 0 | Yes | 122 | No |
| 6/8/2016 | Málaga | 36.73705 | -4.40486 | 1 | Yes | 42 | Yes | 39 | Yes |
| 6/8/2016 | Málaga | 36.73705 | -4.40486 | 1 | Yes | 18 | No | 0 | No |
| 6/8/2016 | Málaga | 36.73705 | -4.40486 | 1 | Yes | 57 | No | 0 | No |
| 6/8/2016 | Málaga | 36.73705 | -4.40486 | 1 | Yes | 7 | No | 0 | No |
| 6/8/2016 | Málaga | 36.73705 | -4.40486 | 1 | Yes | 169 | No | 0 | No |
| 6/8/2016 | Málaga | 36.73705 | -4.40486 | 1 | Yes | 89 | No | 0 | No |
| 6/8/2016 | Málaga | 36.73705 | -4.40486 | 1 | No | 0 | No | 0 | No |
| 6/9/2016 | Málaga | 36.57263 | -4.65161 | 4 | No | 0 | No | 0 | No |
| 6/9/2016 | Málaga | 36.57265 | -4.65656 | 4 | Yes | 37 | No | 0 | No |
| 6/9/2016 | Málaga | 36.57265 | -4.65656 | 1 | No | 0 | No | 0 | No |
| 6/11/2016 | Málaga | 36.75319 | -4.49537 | 1 | Yes | 104 | No | 0 | No |
| 6/11/2016 | Málaga | 36.75319 | -4.49537 | 3 | Yes | 50 | No | 0 | No |
| 6/11/2016 | Málaga | 36.75319 | -4.49537 | 2 | Yes | 95 | Yes | 8 | Yes |
| 6/12/2016 | Málaga | 36.72919 | -4.29747 | 2 | Yes | 61 | No | 0 | No |
| 6/12/2016 | Málaga | 36.72922 | -4.29754 | 1 | No | 0 | Yes | 127 | No |
| 6/13/2016 | Cádiz | 36.31301 | -5.8865 | 3 | Yes | 32 | No | 0 | No |
| 6/13/2016 | Cádiz | 36.31301 | -5.8865 | 3 | Yes | 57 | Yes | 155 | Yes |
| 6/13/2016 | Cádiz | 36.31301 | -5.8865 | 4 | Yes | 69 | Yes | 21 | Yes |
| 6/13/2016 | Cádiz | 36.31301 | -5.8865 | 3 | Yes | 118 | No | 0 | No |
| 6/13/2016 | Cádiz | 36.32299 | -5.88251 | 1 | Yes | 109 | No | 0 | No |
| 6/14/2016 | Cádiz | 36.22979 | -5.89206 | 3 | Yes | 29 | No | 0 | No |
| 6/14/2016 | Cádiz | 36.22979 | -5.89206 | 3 | Yes | 112 | No | 0 | No |
| 6/14/2016 | Cádiz | 36.22979 | -5.89206 | 4 | Yes | 193 | Yes | 6 | Yes |
| 6/14/2016 | Cádiz | 36.22979 | -5.89206 | 3 | Yes | 108 | Yes | 20 | Yes |
| 6/14/2016 | Cádiz | 36.22979 | -5.89206 | 3 | Yes | 35 | Yes | 16 | Yes |
| 6/14/2016 | Cádiz | 36.22979 | -5.89206 | 2 | Yes | 63 | Yes | 311 | Yes |
| 6/14/2016 | Cádiz | 36.22979 | -5.89206 | 2 | Yes | 61 | Yes | 34 | Yes |
| 6/14/2016 | Cádiz | 36.22979 | -5.89206 | 3 | No | 0 | No | 0 | No |
| 6/14/2016 | Cádiz | 36.22979 | -5.89206 | 2 | No | 0 | No | 0 | No |
| 6/14/2016 | Cádiz | 36.22979 | -5.89206 | 2 | No | 0 | No | 0 | No |
| 6/14/2016 | Cádiz | 36.22979 | -5.89206 | 1 | No | 0 | Yes | 123 | No |
| 6/14/2016 | Cádiz | 36.22979 | -5.89206 | 1 | No | 0 | Yes | 18 | No |
| 6/14/2016 | Cádiz | 36.22979 | -5.89206 | 1 | No | 0 | Yes | 272 | No |
| 6/14/2016 | Cádiz | 36.22979 | -5.89206 | 1 | No | 0 | Yes | 248 | No |
| 6/14/2016 | Cádiz | 36.22979 | -5.89206 | 1 | No | 0 | Yes | 198 | No |
| 6/14/2016 | Cádiz | 36.22979 | -5.89206 | 3 | No | 0 | Yes | 210 | No |
| 6/15/2016 | Cádiz | 36.27643 | -6.08103 | 2 | Yes | 32 | Yes | 13 | Yes |
| 6/15/2016 | Cádiz | 36.27643 | -6.08103 | 3 | No | 0 | No | 0 | No |
| 6/15/2016 | Cádiz | 36.27643 | -6.08103 | 3 | No | 0 | No | 0 | No |
| 6/15/2016 | Cádiz | 36.27643 | -6.08103 | 1 | No | 0 | Yes | 119 | No |
| 6/15/2016 | Cádiz | 36.27643 | -6.08103 | 1 | No | 0 | Yes | 95 | No |
| 6/15/2016 | Cádiz | 36.27643 | -6.08103 | 2 | No | 104 | No | 0 | No |
| 6/15/2016 | Cádiz | 36.27721 | -6.08213 | 3 | Yes | 45 | No | 0 | No |
| 6/15/2016 | Cádiz | 36.27721 | -6.08213 | 1 | Yes | 17 | Yes | 13 | Yes |
| 6/15/2016 | Cádiz | 36.27721 | -6.08213 | 1 | No | 0 | No | 0 | No |
| 6/16/2016 | Cádiz | 36.29552 | -6.0748 | 2 | Yes | 129 | No | 0 | No |
| 6/16/2016 | Cádiz | 36.29552 | -6.0748 | 1 | Yes | 40 | No | 0 | No |
| 6/16/2016 | Cádiz | 36.29552 | -6.0748 | 2 | Yes | 67 | No | 0 | No |
| 6/16/2016 | Cádiz | 36.29552 | -6.0748 | 1 | Yes | 130 | No | 0 | No |
| 6/16/2016 | Cádiz | 36.29552 | -6.0748 | 2 | Yes | 113 | No | 0 | No |
| 6/16/2016 | Cádiz | 36.29552 | -6.0748 | 1 | Yes | 43 | Yes | 1 | Yes |
| 6/16/2016 | Cádiz | 36.29552 | -6.0748 | 1 | No | 0 | Yes | 172 | No |
